# Building Better Environmental Risk Assessments

**DOI:** 10.3389/fbioe.2015.00110

**Published:** 2015-08-06

**Authors:** Raymond Layton, Joe Smith, Phil Macdonald, Ramatha Letchumanan, Paul Keese, Martin Lema

**Affiliations:** ^1^Industry Affairs and Regulatory, DuPont Pioneer, Johnston, IA, USA; ^2^Advisor in Regulation, Science and Government (Formerly affiliated with the Office of the Gene Technology Regulator), Canberra, ACT, Australia; ^3^Plant and Biotechnology Risk Assessment Unit, Canadian Food Inspection Agency, Ottawa, ON, Canada; ^4^Department of Biosafety, Ministry of Natural Resources and Environment, Putrajaya, Malaysia; ^5^Office of the Gene Technology Regulator, Canberra, ACT, Australia; ^6^Ministry of Agriculture, Livestock and Fisheries of Argentina, Buenos Aires, Argentina

**Keywords:** genetically engineered crops, GMO, non-target organisms, risk assessment, risk characterization, risk management

## Abstract

Risk assessment is a reasoned, structured approach to address uncertainty based on scientific and technical evidence. It forms the foundation for regulatory decision-making, which is bound by legislative and policy requirements, as well as the need for making timely decisions using available resources. In order to be most useful, environmental risk assessments (ERAs) for genetically modified (GM) crops should provide consistent, reliable, and transparent results across all types of GM crops, traits, and environments. The assessments must also separate essential information from scientific or agronomic data of marginal relevance or value for evaluating risk and complete the assessment in a timely fashion. Challenges in conducting ERAs differ across regulatory systems – examples are presented from Canada, Malaysia, and Argentina. One challenge faced across the globe is the conduct of risk assessments with limited resources. This challenge can be overcome by clarifying risk concepts, placing greater emphasis on data critical to assess environmental risk (for example, phenotypic and plant performance data rather than molecular data), and adapting advances in risk analysis from other relevant disciplines.

## Introduction

Environmental risk assessment (ERA) utilizes a reasoned, structured approach to address uncertainty based on scientific and technical evidence (Wolt et al., 2010). It forms the foundation for regulatory decision-making, which is bound by legislative and policy requirements. It also responds to needs for risk managers to make timely decisions using available resources. The ERA process, which consists of problem formulation, hazard and exposure evaluation, and risk characterization, has been shown to be useful for a broad array of environmental issues.

In problem formulation, policy requirements and protection goals are identified and defined, existing data are gathered and reviewed for utility, conceptual models are developed to identify exposure pathways and potentially affected organisms and/or biological systems, and significant areas of uncertainty are identified. When conducted properly, the problem formulation phase will result in a clear identification of critical uncertainties and a plan to address those uncertainties. If it is determined in problem formulation that additional data are needed to make a decision on whether the risk is acceptable or unacceptable, then hypothesis-based research plans are developed to reduce any remaining critical uncertainties (Raybould, 2006; Wolt et al., 2010).

During risk characterization, research plans are implemented to gather hazard and exposure data. Many regulatory systems utilize a tiered approach to increase the efficiency of data collection for potential hazards (Romeis et al., 2006). Early tier tests conducted with surrogate species use exposure levels, which exceed expected environmental conditions to identify potential taxa of concern. These taxa can then be tested using more realistic testing scenarios. Risk analysis combines exposure and hazard data to evaluate the potential for the occurrence of unacceptable adverse effects under real world conditions. The risk analysis report must be transparent as to how the hazard and exposure data were gathered, what assumptions were made, and how risk was determined.

A critical task in the ERA process is clearly separating essential information from data of marginal relevance or value for evaluating risk [OGTR (Office of the Gene Technology Regulator), 2013]. Some data may be of high quality, but not relevant to the questions being asked in the assessment. Other data may be of low or uncertain quality and not useable for the assessment. However, data that are not directly applicable, fully conclusive, or of lower quality may at times be useful as part of a weight of evidence approach that can contribute to understanding risk. Data may also need to be gathered because of legislative requirements or regulatory guidelines. And lastly, at times data are gathered in response to public pressure or simply in the personal interest of risk analysts or managers. The challenge is to understand which data are needed to reduce critical uncertainties in the risk assessment. The ideal process to build a “fit for purpose” risk assessment would be one that gathers and evaluates only the data required to make the risk decision. For best results, this information should be communicated in a fashion to facilitate decision-making, communication of the rationale supporting the decision, and implementation of any future actions.

While the steps of risk assessment are constant, the underlying process must be adapted to accommodate different genetically modified (GM) organisms being evaluated within a variety of legal and cultural scenarios. Examples of adaptation are evident in the following case studies: evaluation of a novel trait in Canada, evaluation of a GM insect in Malaysia, and a shift from evaluation of imported events to cultivation of locally developed GM crops in Argentina. In each case, the basic approach remained the same and steps were taken to make the assessment as efficient as possible while still being responsive to local needs.

## Case Study: Plants with Novel Traits in Canada

Under current regulations, Canada evaluates all “plants with novel traits” (PNTs) – those plants into which one or more traits have been intentionally introduced, the introduced trait is new to the cultivated crop in Canada, and the trait has the potential to affect the use and safety (environment, human, animal) of the plant (Macdonald and Yarrow, 2003; Macdonald, 2011). This evaluation includes but is not limited to GM crops. The focus in the assessment is a specified use of a certain product, not the process used to develop the product. A pre-market evaluation may be triggered by breeding techniques used to create new crop varieties, for example, conventional breeding including the use of chemical mutagens for variety development, new plant breeding techniques, such as genome editing with TALENs or CRISPRs, and genetic engineering. The pre-market assessment is conducted under the authority of three acts in Canada: the Seeds Act is the authority under which environmental assessments are conducted; the Feeds Act is the authority for the livestock feed assessments; and the Food and Drugs Act provides the authority for food assessment. The overarching authority for biotechnology regulation in Canada derives from the Canadian Environmental Protection Act. The biotechnology regulatory process is designed to be transparent, consistent with international standards, conducted on a case-by-case basis, and science. An important regulatory goal is to ensure that the regulatory system has adequate safeguards to protect human and livestock health and the environment, but does not prevent Canadians from accessing the potential benefits of the products of biotechnology. The challenge has been and continues to be to create a regulatory system that can efficiently evaluate and make decisions on a large number and wide range of potential novel organisms, learn from experience, and adapt as technology evolves. As a consequence, safety assessments that meet regulatory needs for PNTs focus on the novel trait and the “need to know” factors associated with critical uncertainties in the safety assessment. The overall safety assessment process remains the same, whatever breeding technologies were used to develop the PNT. During problem formulation it is determined if there are realistic routes to harm and what data are required to test hypotheses that allow one to predict the likelihood and severity of harm to assessment endpoints associated with protection goals. The focus of the safety assessment is also determined by the end use of the PNT. For most PNTs, a review will be triggered for all relevant groups of risk assessors. For some PNTs, only certain groups within the agencies review the novel crop. Examples are (1) a novel turfgrass would undergo an environmental assessment and may trigger a feed assessment, but not be evaluated as a novel food; (2) a GM forage crop would be evaluated as a novel feed and would undergo an environmental assessment, but would not likely be evaluated as a novel food; (3) a crop, such as GM cotton, would undergo a novel food and feed assessment, but the environmental assessment would be minimal as it would only consider incidental (and transitory) exposure because cotton is neither cultivated nor persistent in Canada.

Problem formulation is used to focus the comparative safety assessment on the differences between the PNT and the conventional counterpart. In this process, a reasonable comparator, usually the unmodified counterpart to the PNT, is chosen and differences between the PNT and the comparator are considered as to whether they represent a probable route to a potential harm. Data are used to evaluate effects on assessment endpoints that relate to the risk hypotheses. The identification of potential hazards is structured under five broad considerations: weediness potential, gene flow to related species that could become a weed, potential for the PNT to become a plant pest, impact on non-target organisms, and impact on biodiversity. The amount of data that are required to evaluate the effects on assessment endpoints is determined on a case-by-case basis. If the PNT is familiar and the change is not very complex or is similar to other crops that have already been considered, then the data requirements can be reduced and linkages to existing data can be made. After the PNT is authorized for unconfined release, it can be used in traditional breeding programs to develop new varieties without additional environmental assessment, though there is a requirement to notify the CFIA if any new information regarding the environmental safety of the PNT becomes available.

It is apparent that the case-by-case comparative approach has served very well for the risk assessment of GM crops in Canada: approximately 100 PNT have received commercial authorization since 1996. This process has been shown to be useful to evaluate and reliably predict the potential effects of the intended change and the potential effects of unintended changes. It takes into account previous experience with insertional effects, normal plant genetic variability, and change over time (including rate of spontaneous mutations, chromosome loss, etc.).

Worldwide, there is growing familiarity with GM crops, foods, and feeds. Coupled with a growing body of research on genetic engineering and fueled by the rapid advancements in molecular analysis techniques, this has provided an unprecedented understanding of plant genomes and genetic change. Canada continues to refine its regulatory process. For example, regulators considered that molecular characterization of a GM crop could be an important component of the risk assessment for environmental, food, and feed safety and so established that component as an area for refinement. A project was initiated to evaluate the potential outcomes of inserting a new gene into a plant in the context of the changes that will occur in a plant genome either spontaneously or through other more conventional forms of plant breeding. This project resulted in a recent publication that examines insertional mutagenesis from transgene insertion in context with the normal changes that can occur in a plant genome and concluded that the risk level for insertional effects is similar to other genetic changes in plants (Schnell et al., 2015). This information helps risk managers place any effects from insertions into the proper context when making regulatory decisions. Further refinements in the regulatory oversight of PNTs are expected as Canada continues to ensure a fit for purpose approach.

## Case Study: Genetically Modified Mosquitoes in Malaysia

Dengue, carried by *Aedes aegypti*, is a significant public health issue with approximately 50–100 million infections occurring each year, resulting in over 20,000 deaths (WHO, 2012). A control strategy was developed where GM male mosquitoes (Strain OX513A) that are dependent on regular doses of tetracycline for survival would be released into the environment where they would mate with wild-type females and produce larvae that would not develop into adults (Lacroix et al., 2012). The GM males would then die within days after release. The system worked well under laboratory conditions; however, additional data were needed since significant differences in behavior in natural conditions could affect the efficacy of the control strategy. A study was designed where approximately 6000 modified and 6000 unmodified male mosquitoes would be released into an uninhabited area of Malaysia. Each of the mosquitoes would be marked using a fluorescent marker and the OX513A mosquitoes could also be identified using PCR. Sampling stations would be located at various distances from the release point and samples taken over a period of time.

Three major requirements had to be met before the study could be conducted: an ERA was needed for the release location, Malaysian regulations required an extensive public communication program, and a final regulatory decision to approve the field trial was needed quickly (<180 days) to meet study timelines. The Genetic Modifications Advisory Committee gathered and evaluated existing data and conducted the ERA. Data were available from laboratory studies, a previously conducted semi-field (caged) mark-recapture study using fluorescent dye, a literature review, a field release study conducted previously in another environment in the Cayman Islands (Harris et al., 2011), and a review of the qualifications of the research team. Previous risk-benefit analyses for this technology that indicated that the potential benefits outweigh the theoretical environmental risk were reviewed for their applicability to the proposed Malaysian study (Beech et al., 2009; Vasan, 2010; Morris, 2011; Reeves et al., 2012). No major risks were identified in the science-based ERA and this was communicated to the risk managers (National Biosafety Board; NBB).

A public communication program was designed to gather information about public concerns related to environmental safety and human health. Challenges in working with the public included an expectation of zero risk and also misconceptions and speculations as to what might happen in the trial. These challenges were addressed via extensive communication with various public groups and media representatives. As summarized by Subramaniam et al. (2012), the public communication effort included (1) multiple public announcements in national newspapers in two languages, (2) direct invitations to nine environmental non-government organizations (NGOs) for comments and invitations to face-to-face meetings, (3) a 30-day public comment period, (4) posters in four languages were placed in local areas near the study site, and (5) meetings organized with both local government councils and local residents. The information gathered from these communication efforts was then supplied to the NBB for evaluation. Some groups were supportive of the technology, some groups were neutral, and some were concerned with the use of GM technology to control dengue.

Once the science-based ERA and societal input were available, the NBB evaluated the information and made a decision to move ahead with the field release study subject to various restrictions (single uninhabited site, increased efforts to make sure only male mosquitoes were released, and fogging the site with insecticides at the end of the study). Information describing the decision and the study was then placed on the NBB website and a press conference held. By focusing on relevant issues, the ERA was conducted quickly, a public communication campaign was completed effectively, a regulatory decision was made that took into account both scientific information and societal influence. The study was conducted successfully and results of the study indicated that dispersal distances and longevity were similar between the two strains. This information can then be used in future ERAs as the GM mosquito project advances.

## Case Study: Redesign of the Assessment Framework in Argentina

Argentina was one of the first countries to approve the cultivation of GM crops and is currently the third largest producer of GM crops in the world – in 2012 a total of 24.4 million hectares were planted with soybean, maize, and cotton (James, 2013). Regulation of GM crops first began in 1991 under the National Advisory Commission on Agricultural Biotechnology. While in some ways similar to other countries, the Argentinian regulatory process was influenced by locally defined protection goals and societal attitudes. This resulted in a regulatory process with some differences compared to the countries where the technology was originally developed or where the regulatory process was designed to evaluate imported grain. In the early 1990s, evaluations in Argentina were designed to assess the potential environmental risk associated with research trial plots. The first commercial release of a glyphosate tolerant soybean was authorized in 1996 and was followed in 1998 by GM maize and cotton. As the number of applications increased, the Biotechnology Office was established in 2004, and then in 2008 the Biotechnology Directorate was established. The changes in the regulatory structure reflected the history of GM crops in Argentina. GM crops originally cultivated in Argentina were developed based on technologies developed overseas, which were adopted into local agricultural production. This means that, in most cases, a significant proportion of regulatory data used in making decisions in Argentina was generated for submissions made previously in other countries. Over time, however, the balance has shifted from crops developed using imported technologies toward locally developed crops. This shift was helped in part by support from the government in terms of incentives to create partnerships to support new biotech startup companies. The production of many new GM crops and varieties developed locally are now moving forward rapidly. Drought tolerance, virus and insect resistance, and herbicide tolerance has been engineered into biotech sugarcane, maize, soybean, wheat, potatoes, and several events are in a medium or advanced stage of deregulation in Argentina.

The challenge over the past 25 years has been to evaluate an increasing number of regulatory submissions on increasingly wider types of crops and varieties. Organizational changes could only go so far in terms of being able to respond to the increased rate and complexity of submissions. In 2012, a new regulatory framework was implemented with a goal of reducing the time needed to evaluate submissions. This change necessitated not just an organizational change, but an increased focus on collecting and evaluating information in a more efficient manner. Examples of changes in procedure and regulatory philosophy include (1) originally crops were expected to be substantially equivalent, but now substantial equivalence is used as a tier in the risk assessment and new criteria are in place to evaluate crops that are not substantially equivalent; (2) previously any gene flow was viewed as directly harmful, now gene flow is regarded as a potential pathway to harm if a definite hazard has been identified under a proper risk hypothesis; (3) under the new framework, requirements vary on a case-by-case basis (e.g., data requirements for contained greenhouse use are different than those for open cultivation); and (4) new regulations and assessment criteria have been developed for special cases like stacks, RNAi, and NBT (new breeding technologies) crops. This focus on how to gather data in the most efficient possible way and to use only data that are really needed to ensure biosafety and make a regulatory decision has decreased the amount of time needed to evaluate a submission and provide the required information to risk managers (Lema, 2014).

## Conduct of ERA with Limited Resources

One common factor for ERAs wherever they may be conducted is limited resources, such as time, funding, and expertise. Temporal limits may be related to commercial development timelines, agronomic needs (e.g., response to a pest outbreak), or legislative or procedural limits within a regulatory agency. Funding limitations are common in industry, academia, and government. Limitations on expertise continue in many areas of the world for several reasons. Staffing may be limited by available funds, facilities, or lack of experts available within a certain geography or technical area. Also, little may be known about a crop, an agro-ecosystem, target and non-target organisms, or a new trait.

A common response is a call for additional resources to conduct ERAs in low-income countries. However, the considerable disparity of resources between high- and low-income countries is not necessarily reflected in the quality of the risk assessments. The availability of better resources in high-income countries has supported the demand for ever-increasing volumes of data, creating unrealistic expectations, and misleading benchmarks for ERAs in low-income countries. In some cases, there is a desire to identify every change that results from the genetic modification or to continue collecting data until all uncertainty is eliminated (an unattainable goal). This approach neglects the value and importance of fit for purpose ERA where resource utilization is driven by focusing on what is needed to make a sound risk management decision.

A regulatory submission typically contains a large amount of data, including general information about the crop, molecular and protein characterization, composition, response to diseases and pests, spectrum of activity for insecticidal traits, and information on potential weediness and invasiveness. These data vary widely in their importance in terms of helping to make a decision regarding environmental risk. For example, the phenotype of a plant (characteristics that affect establishment, reproduction and dispersal, or potential harm) is far more useful than molecular data, which rarely contribute to the identification of important risks.

Indeed, the risk assessment should clearly distinguish important risk from minimal or trivial risk, so that the degree of consideration is aligned with the level of risk. For example, guidance on the identification of significant risks can be obtained from experience with conventional breeding outcomes of equivalent traits (e.g., insect resistance, herbicide tolerance, or stress tolerance), and by incorporating established weed risk assessment techniques for determining risk from any type of plant (Keese et al., 2014). Simple decision-making tools, such as checklists, decision trees, and concept mapping, can be used to support a structured approach to risk assessment in order to clearly communicate reasoning and acknowledge remaining uncertainty (Keese, 2013).

## Discussion and Conclusion

Beginning the risk assessment process with problem formulation is key to focusing on essential data (Wolt et al., 2010). The first step in problem formulation is to define the context of the risk assessment – understand the protection goals, summarize what is already known about the crop and trait, and define the environment for the assessment. Well-defined protection goals will greatly assist the risk assessor. For example, if the protection goal is to protect certain species at a defined level of exposure, then this focuses the assessment process on a narrow range of hazard and exposure scenarios. Useful assessment endpoints can be established and potential endpoints that would cost resources but not provide useful information can be eliminated. The level of precision required can be determined in advance to avoid spending time and energy in an attempt to quantify with high precision if a decision will be made based on the basis of whether or not a threshold is exceeded. Summarizing the currently available information relevant to the defined protection goals makes sure that time is not spent developing answers to questions that have already been answered elsewhere. Definition of the environment allows the risk assessor to focus efforts on answering questions for the case at hand – so that, for example, if the assessment is for imported grain that will be processed near a port facility, then the assessor does not spend time answering “what if” questions related to widespread long-term cultivation of that crop. The second step in problem formulation is to develop an assessment plan. This plan uses a conceptual model to identify and develop testable hypotheses to address areas of critical uncertainties. An analysis plan is then designed that can be used to continually maintain focus on the areas most important to making the risk management decision. Perhaps the most important thing to remember during the risk assessment is that the goal is not to gather information, but rather to test relevant hypotheses to provide specific information that will be useful to the risk manager.

Risk assessors and managers involved in each of the case studies described above faced different cultural, legislative, scientific, and resource challenges. In each example, ways were found to focus the assessment process on obtaining information important to making the risk management decision and to reduce use of valuable resources on lower-value data and activities. In Canada, there was a focus on defining harm and areas of critical uncertainty and then recognizing which groups within the government agencies could respond most effectively. In Malaysia, it was recognized early in the process that a successful outcome would only occur if an extensive public communications effort was developed in parallel with the ERA. In Argentina, the choice was made to move away from a single process to a case-by-case approach; a new framework was developed to evaluate crops and traits and focus on data in each case to make a risk management decision. In each case, efficiency of the regulatory process was greatly increased. Understanding that not all data are of equivalent value and focusing on data that are most relevant to making a risk management decision can alleviate some resource constraints by making the risk assessment and risk management process more efficient. Better ERAs in both high- and low-income countries can be built by focusing on: information that is relevant to identifying important risk (typically phenotypic data); applying a degree of effort that is proportional to the level of risk; and using effective processes to reach sound regulatory decisions.

## Author Contributions

This paper is based on the opening plenary session entitled “Advancing ERA – Fit for Purpose” of the 13th International Symposium on the Biosafety of Genetically Modified Organisms held in November 2014 in Cape Town, RSA. The views expressed in this paper are the views of the authors as individuals and experts in the field, and do not necessarily represent those of the organizations where they work.

## Conflict of Interest Statement

The authors declare that there are no commercial or financial relationships that could be construed as a potential conflict of interest in terms of the topics discussed in this paper.

## References

[B1] Beech C. J. Nagaraju J. Vasan S. S. Rose R. I. Othman R. Y. Pillai V. (2009). Risk analysis of a hypothetical open field release of a self-limiting transgenic *Aedes aegypti* mosquito strain to combat dengue. Asia Pac. J. Mol. Biol. Biotechnol. 17, 99–111.

[B2] Harris A. F. Nimmo D. McKemey A. R. Kelly N. Scaife S. Donnelly C. A. (2011). Field performance of engineered male mosquitoes. Nat. Biotechnol. 29, 1034–1037. 10.1038/nbt.2019 22037376

[B3] James C. (2013). Global Status of Commercialized Biotech/GM Crops: 2013. Ithaca, NY: ISAAA. ISAAA Brief No. 46.

[B4] Keese P. (2013). “Building an effective biosafety regulatory system: the nuts and bolts,” in Collection of Biosafety Reviews, Vol. 8 (Trieste: International Centre for Genetic Engineering and Biotechnology), 10–39.

[B5] Keese P. K. Robold A. V. Myers R. C. Weisman S. Smith J. (2014). Applying a weed risk assessment approach to GM crops. Transgenic Res. 23, 957–969. 10.1007/s11248-013-9745-0 24046097 PMC4204014

[B6] Lacroix R. McKemey A. R. Raduan N. Kwee Wee L. Hong Ming W. Guat Ney T. (2012). Open field release of genetically engineered sterile male *Aedes aegypti* in Malaysia. PLoS ONE 7:e42771. 10.1371/journal.pone.0042771 22970102 PMC3428326

[B7] Lema M. (2014). Directorate History. Available at: http://www.minagri.gob.ar/site/agregado_de_valor/biotechnology/10-DIRECTORATE/index.php

[B8] Macdonald P. (2011). The Canadian experience with novel herbicide tolerant canola. J. Verbr. Lebensm. 6(Suppl. 1), S91–S97. 10.1007/s00003-011-0693-412595139

[B9] Macdonald P. Yarrow S. (2003). Regulation of *Bt* crops in Canada. J. Invertebr. Pathol. 83, 93–99. 10.1016/S0022-2011(03)00059-4 12788275

[B10] Morris E. J. (2011). Open field release of a self-limiting transgenic *Aedes aegypti* mosquito strain to combat dengue – a structured risk-benefit analysis. Asia Pac. J. Mol. Biol. Biotechnol. 19, 107–110.

[B11] OGTR (Office of the Gene Technology Regulator). (2013). Risk Analysis Framework 2013. Commonwealth of Australia. Available at: http://www.ogtr.gov.au/internet/ogtr/publishing.nsf/Content/raffinal5-toc/$FILE/raffinal5_2.pdf

[B12] Raybould A. (2006). Problem formulation and hypothesis testing for environmental risk assessments of genetically modified crops. Environ. Biosafety Res. 5, 119–125. 10.1051/ebr:2007004 17445509

[B13] Reeves R. G. Denton J. A. Santucci F. Bryk J. Reed F. A. (2012). Scientific standards and the regulation of genetically modified insects. PLoS Negl. Trop. Dis. 6:e1502 10.1371/journal.pntd.0001502 22319640 PMC3269433

[B14] Romeis J. Bartsch D. Bigler F. Candolfi M. Gielkens M. Hartley S. (2006). “Moving through the tiered and methodological framework for non-target arthropod risk assessment of transgenic insecticidal crops,” in The 9th International Symposium on the Biosafety of Genetically Modified Organisms, (Jeju Island: Biosafety Research and Environmental Risk Assessment), 64–69. Published by International Society for Biosafety Research, Saskatoon, Canada.

[B15] Schnell J. Steele M. Bean J. Neuspiel M. Girard C. Dormann N. (2015). A comparative analysis of insertional effects in genetically engineered plants: considerations for pre-market assessments. Transgenic Res. 24, 1–17. 10.1007/s11248-014-9843-7 25344849 PMC4274372

[B16] Subramaniam T. S. S. Lee J. L. Ahmad N. W. Murad S. (2012). Genetically modified mosquito: the Malaysian public engagement experience. Biotechnol. J. 7, 1321–1327. 10.1002/biot.201200282 23125042

[B17] Vasan S. S. (2010). Modified insects: risk analysis and public engagement. Asia Pac. J. Mol. Biol. Biotechnol. 18, 237–239.

[B18] WHO. (2012). Global Strategy for Dengue Prevention and Control. Geneva: World Health Organization Available at: www.who.int

[B19] Wolt J. D. Keese P. Raybould A. Fitzpatrick J. W. Burachik M. Gray A. (2010). Problem formulation in the environmental risk assessment for genetically modified plants. Transgenic Res. 19, 425–436. 10.1007/s11248-009-9321-9 19757133 PMC2865628

